# Significant disparity in base and sugar damage in DNA resulting from neutron and electron irradiation

**DOI:** 10.1093/jrr/rru059

**Published:** 2014-07-17

**Authors:** Dalong Pang, Jeffrey S. Nico, Lisa Karam, Olga Timofeeva, William F. Blakely, Anatoly Dritschilo, Miral Dizdaroglu, Pawel Jaruga

**Affiliations:** 1 Department of Radiation Medicine, Georgetown University Hospital, 3800 Reservoir Road, LL Bles, Washington, DC 20007, USA; 2 Radiation Physics Division, National Institute of Standards and Technology, Gaithersburg, MD 20899, USA; 3 Scientific Research Department, Armed Forces Radiobiological Research Institute, Uniformed Services University of the Health Sciences, Bethesda, MD 20889, USA; 4 Biomolecular Measurement Division, National Institute of Standards and Technology, Gaithersburg, MD 20899, USA

**Keywords:** electron LINAC irradiation, ^252^Cf decay fission neutrons, 8-hydroxy-2′-deoxyguanosine, (5′*R*)-8,5′-cyclo-2′-deoxyadenosine, and (5′*S*)-8,5′-cyclo-2′-deoxyadenosine, liquid chromatography–isotope-dilution tandem mass spectrometry, relative biological effectiveness

## Abstract

In this study, a comparison of the effects of neutron and electron irradiation of aqueous DNA solutions was investigated to characterize potential neutron signatures in DNA damage induction. Ionizing radiation generates numerous lesions in DNA, including base and sugar lesions, lesions involving base–sugar combinations (e.g. 8,5′-cyclopurine-2′-deoxynucleosides) and DNA–protein cross-links, as well as single- and double-strand breaks and clustered damage. The characteristics of damage depend on the linear energy transfer (LET) of the incident radiation. Here we investigated DNA damage using aqueous DNA solutions in 10 mmol/l phosphate buffer from 0–80 Gy by low-LET electrons (10 Gy/min) and the specific high-LET (∼0.16 Gy/h) neutrons formed by spontaneous ^252^Cf decay fissions. 8-hydroxy-2′-deoxyguanosine (8-OH-dG), (5′*R*)-8,5′-cyclo-2′-deoxyadenosine (*R*-cdA) and (5′*S*)-8,5′-cyclo-2′-deoxyadenosine (*S*-cdA) were quantified using liquid chromatography–isotope-dilution tandem mass spectrometry to demonstrate a linear dose dependence for induction of 8-OH-dG by both types of radiation, although neutron irradiation was ∼50% less effective at a given dose compared with electron irradiation. Electron irradiation resulted in an exponential increase in *S*-cdA and *R*-cdA with dose, whereas neutron irradiation induced substantially less damage and the amount of damage increased only gradually with dose. Addition of 30 mmol/l 2-amino-2-(hydroxymethyl)-1,3-propanediol (TRIS), a free radical scavenger, to the DNA solution before irradiation reduced lesion induction to background levels for both types of radiation. These results provide insight into the mechanisms of DNA damage by high-LET ^252^Cf decay neutrons and low-LET electrons, leading to enhanced understanding of the potential biological effects of these types of irradiation.

## INTRODUCTION

Ionizing radiation induces a large variety of DNA lesions, including base and sugar lesions, single-strand breaks (SSBs), lesions involving a base and a sugar (i.e. 8,5′-cyclopurine-2′-deoxynucleosides), DNA-protein cross-links, double-strand breaks (DSBs), and clustered damaged sites [1–4]. DNA damage results from the direct or indirect effect of ionizing radiation. Direct effect is a result of energy deposition directly on DNA or its closest hydration layer, whereas indirect effect is due to the interaction of DNA molecules with radiation-induced free radicals generated in water, such as hydroxyl radicals (•OH), hydrated electrons (e_aq_^−^) and H atoms (H^•^) [5]. Hydroxyl radicals react with the constituents of DNA near or at diffusion-controlled rates, causing damage to the heterocyclic DNA bases and to the sugar moiety by a variety of mechanisms [6]. For DNA in aqueous solution, indirect damage predominates in both low- and high-LET (linear energy transfer) radiations [6–10]; however the percentage of damage from indirect effects due to diffusible •OH is reduced with high-LET radiation due to recombination reactions causing decreases in •OH yields and by the presence of •OH scavengers [11]. The fraction of ‘clustered lesions’ formed at high-LET radiation in aqueous DNA solutions is relatively constant for radiation in conditions of high •OH-scavenging capacities, similar to that found in cell-like environments [12, 13].

Comparison of the effects of low- and high-LET radiations on DNA damage contributes to our knowledge of the mechanisms of radiation-induced damage. The effects of heavy ions in causing single- and double-strand breaks are well characterized, whereas effects such as damage to bases and clustered lesions are less well characterized (reviewed in [14]). Neutron-induced DNA strand breaks in aqueous solution have been previously investigated [11, 13, 15, 16]. However, no studies have been reported on neutron-induced base damage and the formation of 8,5′-cyclopurine-2′-deoxynucleosides. If not repaired by DNA repair mechanisms in living organisms, radiation-induced DNA damage may lead to disease processes such as carcinogenesis [16–19].

In the present work, we have investigated the effects of low-LET electron and high-LET neutron irradiations (the latter produced by spontaneous fission neutrons from ^252^Cf decay) on DNA base damage using liquid chromatography–isotope dilution tandem mass spectrometry (LC–MS/MS). The resulting data provide additional insight on neutron- and electron-induction of DNA lesions, and into the physical and chemical mechanisms of neutron- and electron-induced damage to DNA.

## MATERIALS AND METHODS

### Materials

Calf thymus genomic DNA was purchased from Sigma–Aldrich (St Louis, MO). The DNA was diluted in 10-mmol/l phosphate buffer, pH 7.4 at room temperature and aliquoted into 250-µl Eppendorf tubes containing 60 μg DNA each, and irradiated subsequently.

2-amino-2-(hydroxymethyl)-1,3-propanediol (TRIS) (99.9% purity) was purchased from Sigma-Aldrich at a concentration of 30 mmol/l. Nuclease P1, snake venom phosphodiesterase, and alkaline phosphatase were purchased from United States Biological (Swampscott, MA), Sigma Chemical Co. (St Louis, MO) and Roche Applied Science (Indianapolis, IN), respectively. Water and acetonitrile for LC–MS/MS were purchased from Sigma Chemical Co. (St Louis, MO).

### Electron and neutron irradiation

Electron irradiations of DNA were performed in the Department of Radiation Medicine of Georgetown University Hospital on a medical linear accelerator (Varian Trilogy, Palo Alto, CA). The energy of the electron beam was 6 MeV. A 10 × 10 cm^2^ electron cone was used to collimate the electron beam. The source-to-surface distance was set at 100 cm. A 1.2-cm thick water-equivalent plastic plate was placed on top of the Eppendorf tubes containing the DNA samples to provide the necessary dose build-up. During irradiation, the DNA solution was exposed to the ambient air contained in the Eppendorf tubes. Three samples were irradiated at each dose. The linear accelerator had been calibrated to deliver 1 cGy/1MU at this setting using a calibrated ion chamber traceable to the National Institute of Standards and Technology (NIST) in Gaithersburg, MD. At a dose rate of 10 Gy/min, doses of 10, 20, 40, 60 and 80 Gy were delivered to the samples. The uncertainty of dose was < 2%.

Neutron irradiations were performed at the Californium Neutron Irradiation Facility (CNIF) at NIST, using spontaneous fission neutrons from ^252^Cf decay as previously described [20, 21]. To reduce the gamma component of the radiation field, a lead shield 2.1 cm in thickness was placed between the ^252^Cf source and the samples. During irradiation, the DNA solution was exposed to ambient air contained in the Eppendorf tubes. The mean neutron fluence was converted to charged particle dose using the conversion factor of 3.1 × 10^−11^ Gy cm [22]. In this configuration, the gamma ray component is estimated to be 15% based on simulation using Monte Carlo N-Particle Transport Code, Version 5 (MCNP5) [23]. Neutron irradiation times were calculated based on the known ^252^Cf source activity to achieve the planned doses of 10, 20, 40, 60 and 80 Gy, and they were 2.56, 5.13, 10.25, 15.38 and 20.51 d, respectively. Factors affecting the accuracy of delivered doses included source position, sample position, source activity and model used for calculation. The uncertainties associated with these factors were: source position (10%), sample position (4%), source activity (3%), conversion factor (3%) and modeling (3%). Together these factors contribute to a total neutron dose uncertainty of 12%.

### Analysis by LC–MS/MS

**Fig. 1.**
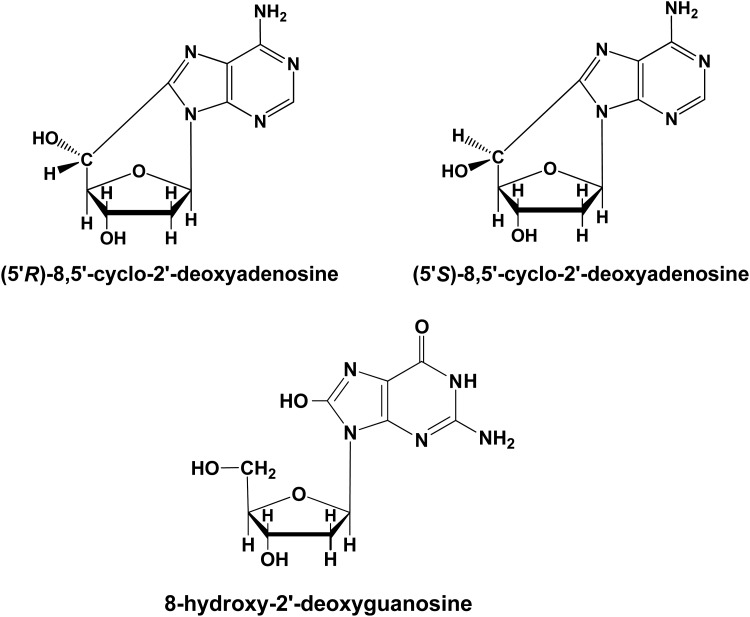
Chemical structures of the *R*-cdA, *S*-cdA and 8-OH-dG.

We used LC–MS/MS to identify and quantify 8-hydroxy-2′-deoxyguanosine (8-OH-dG), (5′*R*)-8,5′-cyclo-2′-deoxyadenosine (*R*-cdA) and (5′*S*)-8,5′-cyclo-2′-deoxyadenosine (*S*-cdA) in DNA samples. Figure 1 shows the chemical structures of these three lesions. Stable isotope-labeled internal standards *R*-cdA-^15^N_5_ and *S*-cdA-^15^N_5_ were prepared and isolated as described [24]. 8-OH-dG-^15^N_5_ was purchased from Cambridge Isotope Laboratories Inc. (Andover, MA). Aliquots of the internal standards were added to 60-μg aliquots of DNA samples (irradiated or control). Samples were dried in a SpeedVac, subjected to enzymatic hydrolysis and analyzed by LC–MS/MS [25].

### Statistical analysis

All experiments were replicated three times. Arithmetic means were calculated, weighting the error. Dose response yields were determined based on linear or exponential fits using least square regression analysis. Relative biological effectiveness ratios were determined at designated product yields based on the initial linear fits to the dose–response relationship. Statistical differences were determined using the Student *t* test at *P* < 0.05.

## RESULTS

**Fig. 2.**
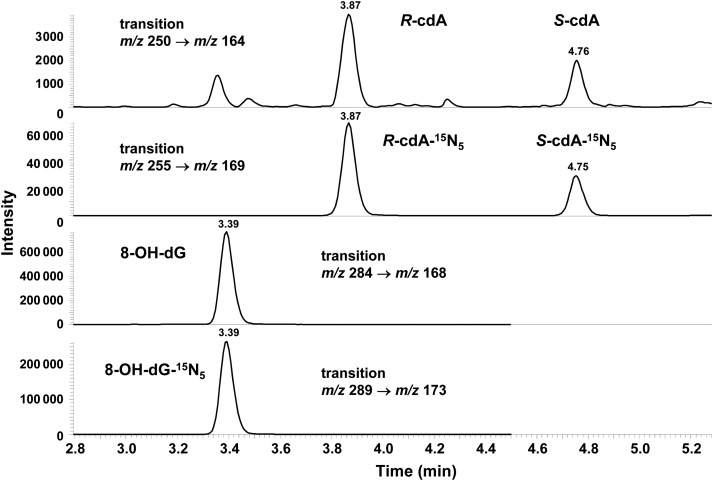
Ion–current profiles of the transitions *m/z* 250 to *m/z* 164 (*R*-cdA and *S*-cdA), *m/z* 255 to *m/z* 169 (*R*-cdA-^15^N_5_ and *S*-cdA-^15^N_5_), *m/z* 284 to *m/z* 168 (8-OH-dG) and *m/z* 289 to *m/z* 173 (8-OH-dG-^15^N_5_) recorded during the LC–MS/MS analysis of a DNA sample irradiated with electrons at dose 40 Gy.

**Fig. 3.**
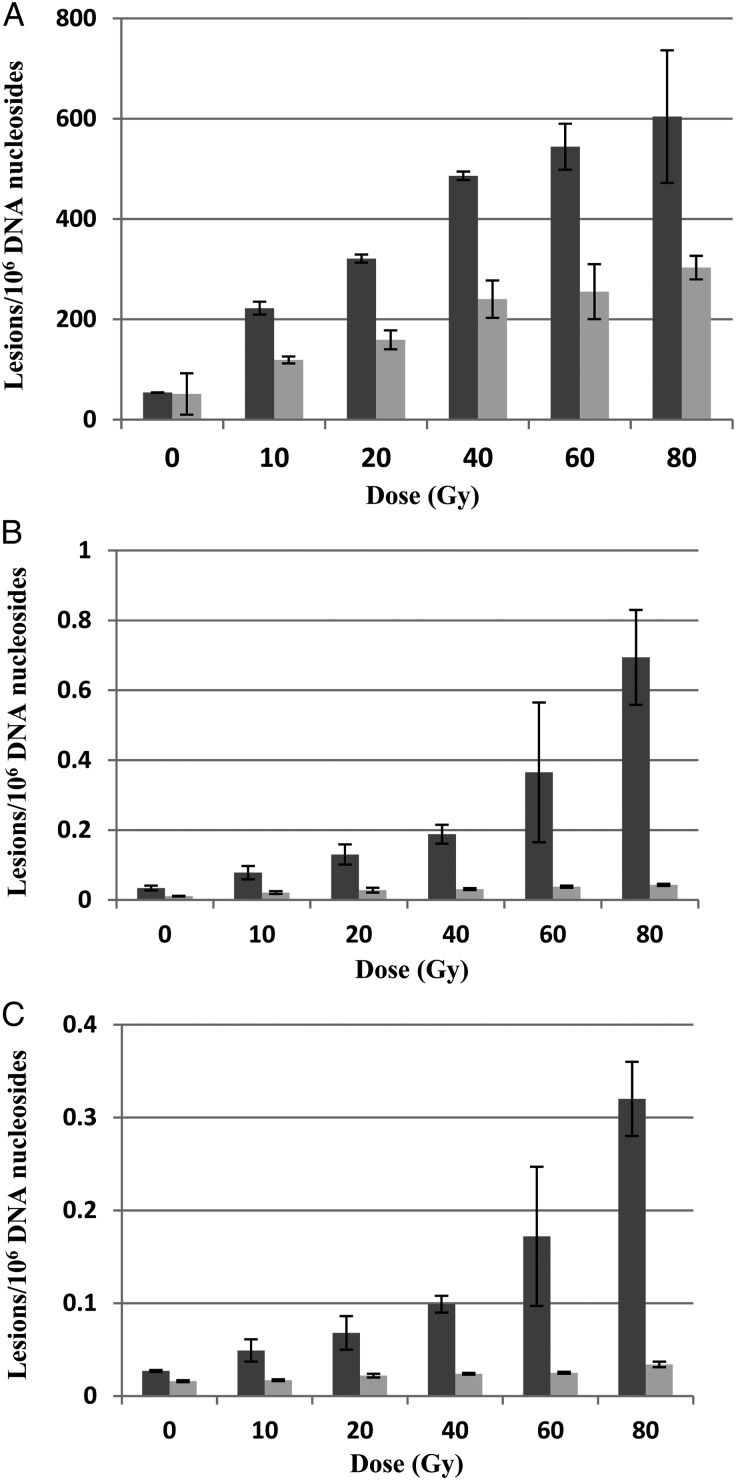
Dose responses of 8-OH-dG (panel **A**), *R*-cdA (panel **B**) and *S*-cdA (panel **C**) induced by irradiation with electrons at 10 Gy/min (dark columns) and neutrons at ∼0.16 Gy/h (light columns). Each datapoint represents the mean of three independent measurements. The uncertainties are standard deviations.

**Fig. 4.**
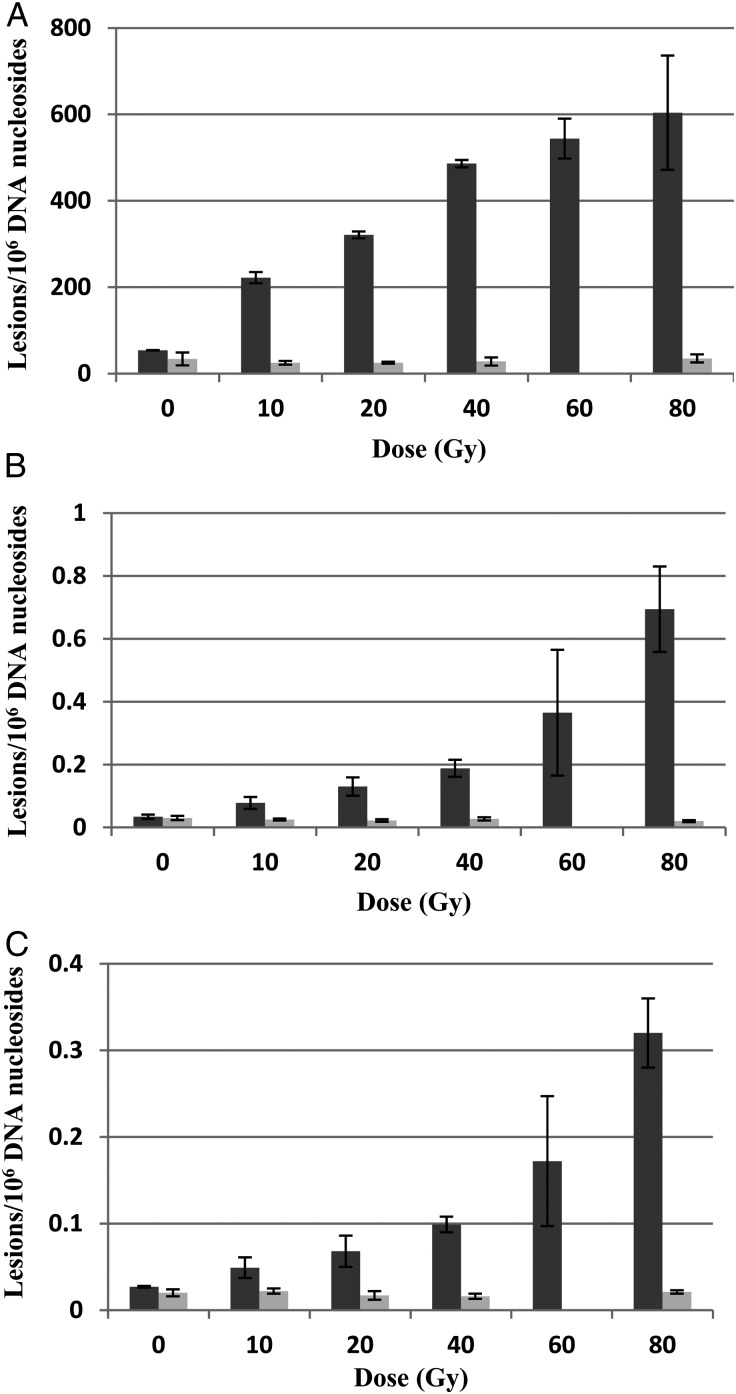
Comparison of electron-radiation-induced 8-OH-dG (panel **A**), *R*-cdA (panel **B**) and *S*-cdA (panel **C**) with (light columns) and without (dark columns) the free radical scavenger TRIS. The missing datapoints at 60 Gy for electron irradiation with TRIS were due to accidental damage to the samples. Each datapoint represents the mean of three independent measurements. The uncertainties are standard deviations.

**Fig. 5.**
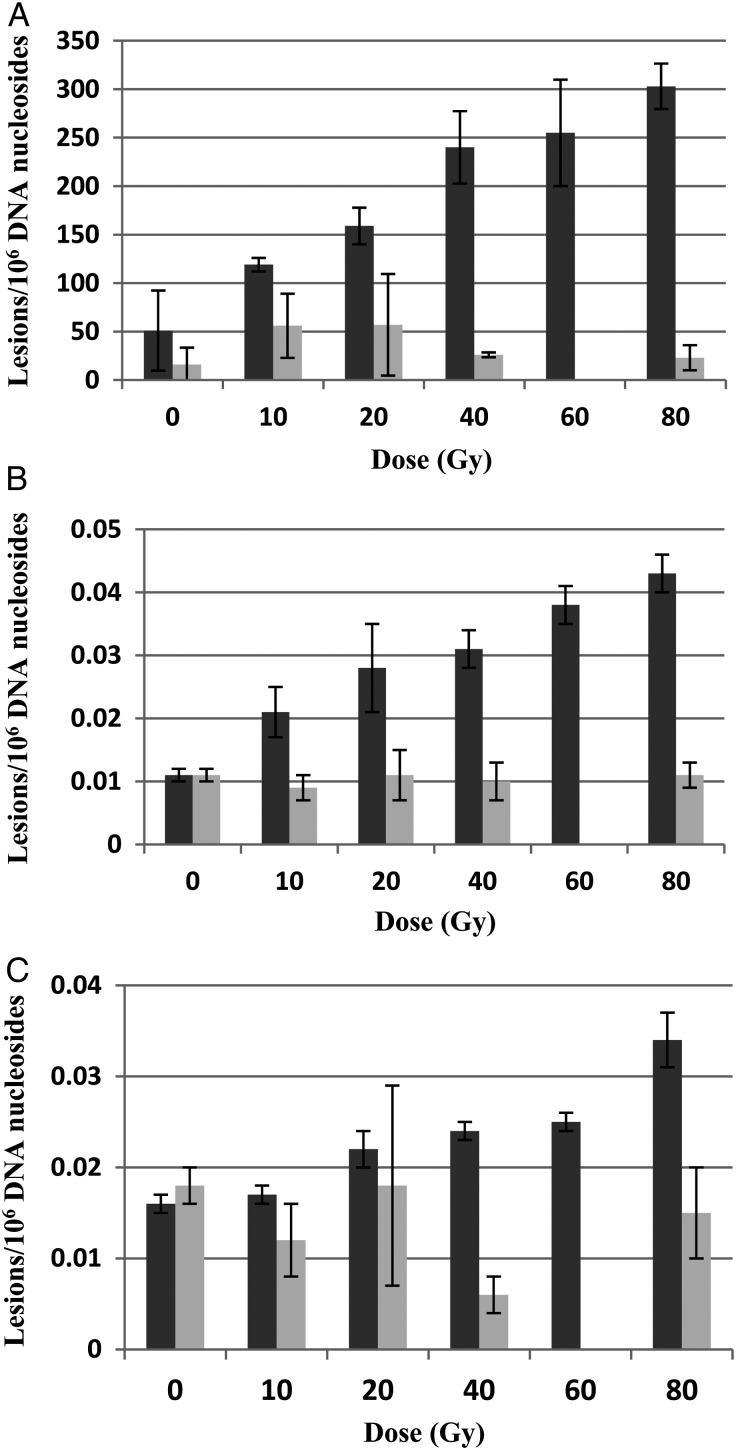
Comparison of neutron-radiation-induced 8-OH-dG (panel **A**), *R*-cdA (panel **B**) and *S*-cdA (panel **C**) with (light columns) and without (dark columns) the free radical scavenger TRIS. The missing datapoints at 60 Gy for neutron irradiation with TRIS were due to accidental damage to the samples. Each datapoint represents the mean of three independent measurements. The uncertainties are standard deviations.

Figure 2 is an illustrative example of the chromatogram of the radiation products produced in a DNA sample irradiated with 40-Gy electrons. The dose responses for the formation of 8-OH-dG, *R*-cdA and *S-*cdA in neutron- and electron-irradiated DNA in 10 mmol/l phosphate buffer are plotted in Fig. 3. Figure 4 shows the quantification of the lesions as a function of dose to electron radiations performed with and without the additional free radical scavenger TRIS at 30 mmol/l present in the solution, while the dose response with and without TRIS for neutron irradiations is given in Fig. 5.

**Fig. 6.**
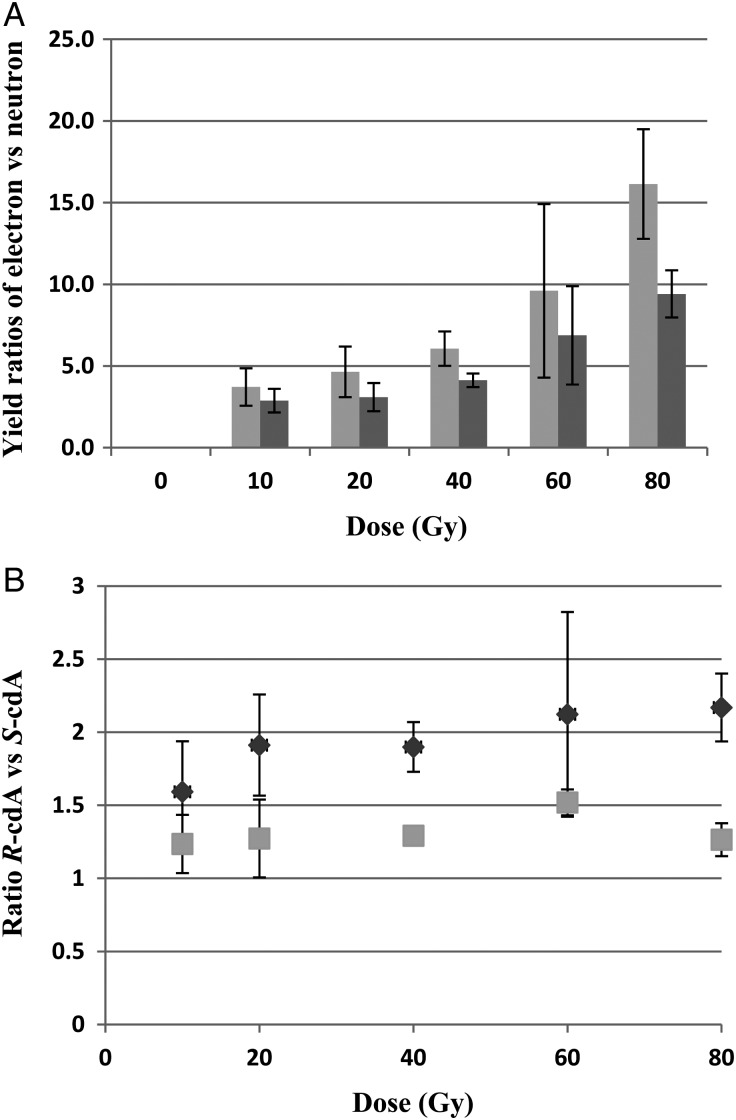
Neutron vs electron effect signatures using relative product yields of *R*-cdA and *S*-cdA. (**A**) Ratios of the yield of *R*-cdA and *S*-cdA lesions induced by electron irradiation relative to neutron irradiation. The light columns represent datapoints for *R*-cdA, and the dark columns represent datapoints for *S*-cdA. (**B**) Ratios of the yield of *R*-cdA relative to *S*-cdA for electron and neutron irradiation. Dark circles represent electrons and light boxes represent neutrons. Each datapoint represents the mean of three independent measurements. The uncertainties are standard deviations.

As shown in Fig. 3, neutron irradiation yielded ∼50% of the 8-OH-dG lesions compared with the yield following electron exposure at the same dose. The trends for both types of radiations are similar in pattern: increasing quickly in the dose range of 0–40 Gy, but less quickly from 40–80 Gy. For *R*-cdA and *S*-cdA induction by electrons, both dose–response curves exhibit exponential increases with dose over the dose range examined; however, the rate of induction of *S*-cdA is approximately half that of *R*-cdA. Neutron induction of both of these lesions is substantially less than the induction of these lesions observed with electrons, and exhibits only small increases with dose. The effects of electron irradiation compared with neutron irradiation on the yields of the *R*-cdA and *S*-cdA lesions irradiated in 10 mmol/l phosphate buffer are shown in Fig. 6. The ratio of *R*-cdA (light bars) and *S*-cdA (dark bars) yields caused by electrons compared with neutrons were plotted as a function of increasing dose (Fig. 6A). These ratios showed a rapid increase with dose; however, the *R*-cdA ratio increased more quickly than that of *S*-cdA. Furthermore, for the *R*-cdA yield, the ratio increased from 3.7 to 16.1 when the dose increased from 10–80 Gy (i.e. nearly exponentially), whereas the yield of *S*-cdA showed a linear increase from 2.9–9.4. The effects of dose on the ability of electron and neutron irradiation to produce either *R*-cdA vs *S*-cdA are illustrated in Fig. 6B. The results are presented as fold changes, shown in Fig. 6B as ratios in log_2_ values. The relative formation of *R*-cdA to that of *S*-cdA is similar for the dose examined. Again, electrons show a trend of producing more *R*-cdA than *S*-cdA. These findings support a signature fingerprint for low-LET electron irradiation (10 Gy/min) vs neutron irradiation (∼0.16 Gy/h) effects based on the relative yields of these tandem lesions, *R*-cdA and *S*-cdA.

## DISCUSSION

The *R*-cdA, *S*-cdA and 8-OH-dG measured in this investigation represent the first experimental data for these DNA lesions being induced by low-LET electron and high-LET neutron irradiation of genomic DNA. SSBs produced by low-LET radiation are reportedly produced some three times more than that induced by fission neutrons and were attributed to the reduced yield of •OH produced by high-LET radiation compared with low-LET radiation [11]. In this report, the number of the 8-OH-dG lesions per unit dose was found to increase with dose for both electron and neutron irradiation; however, at any given dose, fission neutrons from ^252^Cf decay generated half the number of these lesions, confirming a similar •OH mechanism for the generation of SSBs and 8-OH-dG lesions and the reduced capacity of •OH induction by neutrons.

The observed induction rate differences between *R*-cdA and *S*-cdA following electron irradiation reported here are consistent with the results of Jaruga *et al.* with photon (gamma) irradiation, which showed an ∼2-fold difference in induction of *R*-cdA compared with *S*-cdA in calf thymus DNA [26]. This is not surprising, considering both electron and photon irradiation are low-LET radiation. Interestingly, while the relative ratios are consistent, the absolute numbers of lesions per Gy in 10^6^ DNA bases are two orders of magnitude greater in Jaruga *et al.*'s earlier findings. Such a difference can be explained by the conditions under which the samples were irradiated, i.e. in the presence of N_2_O without oxygen (whereas our electron irradiations were conducted in ambient air). Oxygen is known to prevent the formation of 8,5′-cyclopurine-2′-deoxynucleosides because it rapidly reacts with the 5′-centered radical of 2′-deoxyribose of DNA, inhibiting 5′,8′-cyclization (reviewed in [6]). Cadet and colleagues have performed measurements of radiation-induced DNA base lesions using controlled gassing conditions [27–29]. In addition to using ^60^Co γ-rays, they measured eight base lesions caused by high-LET carbon ions using LC–MS/MS and observed a 2-fold less lesion induction by carbon ions compared with photons for both thymidine glycol and 8-OH-dG within the dose range of 90–450 Gy [30]. It should be noted that our observation of a 2-fold reduction in 8-OH-dG by neutrons coincides with their findings with carbon ions, although the measured base lesions are different. The LET of the carbon ions in their experiments varied from 25.2–31.52 keV/µm in the irradiated cell medium. The ^252^Cf fission neutron beam in our experiments had an average energy of 2.1 MeV, with a LET in the same range. Such consistency supports the LET dependence of base lesion induction regardless of the radiation applied. Interestingly, this observation for base lesions is consistent with the induction of SSBs quantified with gel electrophoresis [31].

The substantially lower efficiency of neutron induction of *R*-cdA and *S*-cdA can be addressed qualitatively by the clustered nature of neutron ionization, which results in the dense formation of free radicals within each cluster, but sparsely distributed free radical clusters. The dense free radicals within each cluster have a much shorter range of diffusion and a higher frequency of neutralization via recombination reactions, resulting in a reduced capacity for DNA lesion induction [32, 33].

While there are large differences in *R*-cdA (7-fold) and *S*-cdA (5-fold) induction by electrons and neutrons, the difference in 8-OH-dG is only 2-fold, suggesting differences in the mechanisms underlying the induction of 8-OH-dG from those underlying the induction of *R*-cdA or *S*-cdA (Figs 2 and 5). Previously, the total level of *R*-cdA and *S*-cdA was measured by LC–MS and GC–MS in N_2_O-saturated DNA samples after exposure to ^60^Co γ-radiation, and a yield of 0.65 and 0.70 lesions per 10^6^ DNA bases per Gy, respectively, was found [26]. 8-OH-dG was also measured by LC–MS and GC–MS, and a yield of 7.77–8.06 lesions per 10^6^ DNA bases per Gy was found [26, 34], consistent with our finding of 7.55 lesions per 10^6^ DNA bases per Gy. *R*-cdA, *S*-cdA and 8-OH-dG are typical products of reactions of •OH with DNA components. *R*-cdA and *S*-cdA are tandem lesions and formed by initial abstraction of an H atom by •OH from the 5′-carbon of the sugar moiety, followed by cyclization between the 5′-carbon of the sugar moiety and the 8′-carbon of the base moiety of the same nucleoside, and subsequent oxidation. 8-OH-dG results from •OH addition to the C8-position of guanine followed by oxidation (reviewed in [35]). The rate of reaction of •OH with guanine is much faster than H-abstraction by •OH from the sugar moiety of a nucleoside [35]. Furthermore, the formation of 8-OH-dG is increased by oxygen, whereas oxygen inhibits the formation of cdA. Such mechanistic differences explain the large differences in the induction by ionizing radiations of these two types of lesions (Fig. 3).

The observed exponential increase with dose for *S*-cdA and *R*-cdA at higher doses may be partially explained by the large difference in dose rate for electron and neutron irradiations. At a dose rate of 10 Gy per min, it took only 8 min to deliver 80 Gy for electron irradiation. On the contrary, it took slightly over 20 d to deliver 80 Gy of neutron dose. The high dose rate of electron irradiation may have resulted in a much faster depletion of oxygen contained in the air in the Eppendoff tubes, as well as dynamic differences in the relative ion cluster density, which may have consequently resulted in an increased production of *S*-cdA and *R*-cdA.

Free radical scavengers, e.g. dimethyl sulfoxide (DMSO), glycerol, TRIS, ethanol, etc., have been widely used in DNA damage measurements in the radiation biology community to quantify and distinguish the indirect damage from the direct damage [36]. To examine the roles of direct vs indirect effects of radiation on DNA lesion formation, we added free radical scavenger TRIS to the DNA solutions at a concentration of 30 mmol/l and repeated the irradiation experiments for both electron and neutron irradiation. As shown in Figs 3 and 4, the addition of 30 mmol/l TRIS reduced the induction of all three types of lesions by both electron or neutron irradiation to background levels. To quantify the direct vs indirect effects of radiation, an order of magnitude higher dose may be required, as has been previously demonstrated by Pogozelski *et al.* in their work isolating clustered DNA damage [13]. On the other hand, the strong effect of an •OH scavenger clearly shows the formation of •OH under both irradiation conditions. This is also supported by the fact that *R*-cdA, *S*-cdA and 8-OH-dG are typical products of •OH reactions with DNA. 8-OH-dG may also be formed by the direct effect of ionizing radiations by production of a guanine radical cation followed by reaction with water (addition of •OH) and subsequent oxidation (reviewed in [35]). However, the complete inhibition of the formation of 8-OH-dG by an •OH scavenger excludes the direct effect of radiation under the conditions used in this work.

In conclusion, we have demonsrated that neutrons induce a substantially lower yield of damage in DNA bases and sugars when compared with electron irradiation. The magnitude of the difference depends on the types of lesion measured: a 50% reduction was observed for the base lesion of 8-OH-dG, whereas as high as 7-fold and 5-fold reductions were observed for *R*-cdA, and *S*-cdA, respectively. Taken together, these findings support a characteristic signature for specific high-LET neutron irradiation (∼0.16 Gy/h) vs low-LET electron irradiation (10 Gy/min) effects observed here using the yields of base and tandem DNA lesions (8-OH-dG, *R*-cdA and *S*-cdA).

## FUNDING

Funding to pay the Open Access publication charges for this article was provided by the Department of Radiation Medicine, Georgetown University School of Medicine. No other research funding was utilized for this research.
